# A Single Nucleotide Polymorphism of the Neuropeptide B/W Receptor-1 Gene Influences the Evaluation of Facial Expressions

**DOI:** 10.1371/journal.pone.0035390

**Published:** 2012-04-24

**Authors:** Noriya Watanabe, Mari Wada, Yoko Irukayama-Tomobe, Yousuke Ogata, Natsuko Tsujino, Mika Suzuki, Naoki Furutani, Takeshi Sakurai, Miyuki Yamamoto

**Affiliations:** 1 Comprehensive Human Sciences, University of Tsukuba, Tsukuba, Ibaraki, Japan; 2 Japan Society for the Promotion of Science, Tokyo, Japan; 3 Graduate School of Engineering, Tamagawa University, Machida, Tokyo, Japan; 4 University of Tsukuba Center for Behavioral Molecular Genetics (FIRST Program), Tokyo, Japan; 5 Department of Molecular Neuroscience and Integrative Physiology, Faculty of Medicine, Kanazawa University, Kanazawa, Ishikawa, Japan; 6 Exploratory Research for Advanced Technology Yanagisawa Orphan Receptor Project, Japan Science and Technology Agency, Tokyo, Japan; University of Houston, United States of America

## Abstract

Neuropeptide B/W receptor-1 (NPBWR1) is expressed in discrete brain regions in rodents and humans, with particularly strong expression in the limbic system, including the central nucleus of the amygdala. Recently, Nagata-Kuroiwa et al. reported that *Npbwr1* ^−/−^ mice showed changes in social behavior, suggesting that NPBWR1 plays important roles in the emotional responses of social interactions.

The human *NPBWR1* gene has a single nucleotide polymorphism at nucleotide 404 (404A>T; SNP rs33977775). This polymorphism results in an amino acid change, Y135F. The results of an in vitro experiment demonstrated that this change alters receptor function. We investigated the effect of this variation on emotional responses to stimuli of showing human faces with four categories of emotional expressions (anger, fear, happiness, and neutral). Subjects' emotional levels on seeing these faces were rated on scales of hedonic valence, emotional arousal, and dominance (V-A-D). A significant genotype difference was observed in valence evaluation; the 404AT group perceived facial expressions more pleasantly than did the 404AA group, regardless of the category of facial expression. Statistical analysis of each combination of [V-A-D and facial expression] also showed that the 404AT group tended to feel less submissive to an angry face than did the 404AA group. Thus, a single nucleotide polymorphism of NPBWR1 seems to affect human behavior in a social context.

## Introduction

Recent advances in molecular biology and brain-function imaging technology have enabled us to study genetic influences on emotional responses both behaviorally and physiologically. Nucleotide polymorphisms in monoamine transmitter-related molecules have been extensively studied in relation to emotion and reward systems. For example, in the serotonergic system, genetic variations in the regulatory region of 5-HT transporters (5-HTT) seem to influence the “harm avoidance” trait [1], [2] as assessed by the Tri-dimensional Personality Questionnaire [3] and susceptibility to depression [4] as assessed by NEO personality tests [5]. This variability also causes differences in amygdala activity as shown by functional magnetic resonance imaging (fMRI) when observing emotional visual stimuli [6]. A single nucleotide polymorphism (SNP) in the regulatory region of the 5-HT receptor type 3 gene changes both amygdala activity in response to the stimuli of human faces, and personality trait as assessed by TCI [7], [8].

In the dopaminergic system, Krugel et al. [9] reported that a catechol-O-methyltransferase SNP (V158M) affected learning rate during a reward-based learning paradigm. In their study, the Val/Val group showed a higher learning rate than the Met/Met group, and higher activity in the ventral striatum which was correlated to prediction error.

NPBWR1 (GPR7) is a G-protein-coupled receptor whose ligands were recently identified as neuropeptide W (NPW) and neuropeptide B (NPB) [10], [11], [12], [13] (also see rev. [14]). *NPBWR1* is highly conserved between humans and rodents, and its mRNA is localized in discrete brain regions including the hypothalamus, hippocampus, ventral tegmental area (VTA), and central nucleus of the amygdala (CeA) in rodents [11], [13], [14] and in humans [12]. The distribution of NPBWR1 suggested that it may have a role in the regulation of emotion-related responses that affect autonomic functions.

The amygdala is well known to play a crucial role in emotional and social behaviors [15] (also see rev. [16], [17], [18]), while the hypothalamus plays an important role in emotion, especially aggression (see rev. [19]), as well as in controlling the autonomic nervous system. The distribution of *NPBWR1* in the VTA may also suggest its involvement in the reward system (see rev. [20]).

Hondo et al. [14] reviewed the role of NPBWR1, with more emphasis on the physiological roles of the NPBW system in emotional responses including stress responses and social interactions. Nagata-Kuroiwa et al. [21] behaviorally tested *Npbwr1* ^−/−^ mice, which exhibited abnormal reactions toward an intruder in the resident–intruder test, such that *Npbwr1* ^−/−^ mice showed a shorter latency to initial physical contact with the intruder and longer contact and chasing times with the intruder compared with *Npbwr1* ^+/+^ mice. However, as *Npbwr1* ^−/−^ mice did not show significant differences in an open field test and elevated plus maze test compared with *Npbwr1* ^+/+^, the compulsive behavior toward the intruder did not seem to implicate an increase in general anxiety. Rather, the behavior suggested changes in social interaction.

The human *NPBWR1* gene has a frequent SNP at nucleotide 404 (SNP rs33977775) in the coding region (404A>T). This polymorphism causes an amino acid change (Y135F) in the DRY motif of G-protein-coupled receptors, which has been thought to play an important role in G-protein coupling. We therefore hypothesized that if signal transduction of NPBWR1 is impaired by alteration of the DRY motif, it could influence human behavior as well. To confirm the different response of signal transduction, we first tested whether this SNP could affect the function of human NPBWR1 at the cellular level by transfecting the human NPBWR1 gene into HEK293A cells. As there was a difference in cell line responses by transfection of two different NPBWR1 gene sequences, we presumed that the function of NPBWR1 is different between genotypes (see Results).

As *Npbrw1^−/−^* mice showed abnormalities in social interaction, and NPBWR1 is strongly expressed in the amygdala, which is known to be activated by facial stimuli in human imaging studies, we used pictures of human faces as stimuli to examine behavioral changes caused by genetic difference. To evaluate “subjective” emotional responses to facial visual stimuli, we adopted the Self-Assessment Manikin (SAM) scale developed by Bradley & Lang [22]. SAM is a picture-oriented scale that does not use semantic testing and directly assesses pleasure, arousal and dominance in response to an object or event, and seems to provide sensitivity for distinguishing subtle emotional differences in social interactions between two *NPBWR1* genotypes than the semantic differential scale originally developed by Russell and Mehrabian [23], and Mehrabian [24]. The results showed the suitability of SAM for emotional evaluation by the stimulus of human faces, and elucidated the genotype difference in emotional response in social interaction.

## Materials and Methods

### Effects of SNP on NPBWR1 Function

To express NPBWR1, we subcloned human *NPBWR1* (404T) (n = 6) or *NPBWR1* (404A) (n = 6) cDNA fused with green fluorescent protein (GFP) in pEF4mycHisB (Invitrogen). These constructs, along with pGloSensor-22F cAMP plasmid (Promega), were transfected into HEK293A cells obtained from the RIKEN Cell Bank, using Fugene HD reagent (Roche) following the manufacturer's instructions with modifications. Transfection efficiency monitored by GFP fluorescence was comparable between the two clones (404A, 48.3±11.4% vs. 404T, 47.4±10.2%). To decipher the function of *Npbwr1* with or without the 404 SNP, cells were collected and suspended in Hepes-buffered saline supplemented with 10% FBS and 1% GloSensor cAMP reagent (equilibration medium) and seeded on 96-well plates (45 µl, 2×10^4^ cells per well) and incubated for 2 h. Baseline luminescence was measured, and the reaction was initiated by adding 5 µl ligand (NPB or NPW) solution containing designated ligands and forskolin (final 1 µM). After 30 min of incubation, chemiluminescence was assayed with an Arvo SX plate reader (Wallac). Relative cAMP levels were calculated as the signal to background ratio of luminescence. Fold response was calculated relative to a control sample containing vehicle alone.

### Subjects

For the behavioral studies, 126 volunteers (73 male, 53 female; age, 21.3+2.1 years, mean + SD) participated. The study protocol was approved by the ethics committee of the University of Tsukuba and was performed in accordance with the ethical standards laid down in the 1964 Declaration of Helsinki. All subjects provided written informed consent prior to participating in the experiments. Subjects were students of the University of Tsukuba (first year undergraduate students to second year graduate students). As they had all passed the entrance examination of the University of Tsukuba, their intellectual levels *were* considered to be similar. The subjects' major fields were Humanities (n = 6), Social and International Studies (10), Human Science (7), Life and Environmental Sciences (23), Science and Engineering (27), Informatics (9), Medical Science (27), Health and Physical Education (9), and Art and Design (4).

### SNP genotyping

Oral mucosal cells were collected, and DNA was extracted. The *NPBWR1* gene was amplified by PCR, and SNPs were identified by direct sequencing. The oligonucleotides 5′CGGGGAGCTCATGTGCAA3′ and 5′GCAGCACGACGAGTGTGA3′ were used as PCR primers.

### Statistical analysis by sex and age

To exclude influences other than those of the SNP, we compared the sex and age distribution between genotype groups for each behavioral test. Pearson's χ^2^ test was used for the sex distribution, and two-tailed *t*-test was used for the age distribution.

### Behavioral experiments (Emotional face experiments)

To evaluate social emotional responses, we used photographs of the faces of 16 people displaying four emotions (total, 64). Photographs of angry, fearful, happy, and neutral facial expressions, downloaded with permission from “NimStim Face Stimulus Set” at http://www.macbrain.org/, were used as visual stimuli. The validity and reliability of this set were evaluated by Tottenham et al. [25]. For this study, we chose comparatively high-validity and -reliability stimuli (mean >0.9 for both validity and reliability) from the set.

Participants saw the faces presented on a monitor screen (55 cm from the subject, visual angle 5.8° [height]×5° [width]), and **a)** identified the facial expression (anger, fear, happy and neutral) and **b)** rated each stimulus on scales of hedonic valence, emotional arousal, and dominance (no time limit was imposed for subjects' decisions) according to the Self-Assessment Manikin (SAM) scale [22], which is modified from the three-factor theory of emotion by Russell & Mehrabian [23].

#### a) Facial emotion identification test

Subjects were asked to identify the emotions represented by the facial expressions (anger, fear, happy, and neutral) of the presented stimuli. Total error rate for each expression was compared between the two genotypes by analysis of variance (two-way ANOVA). Error rates for each expression and for combination-specific errors (e.g., mistaking fear for anger, etc.) were statistically tested between the two genotypes using two-tailed *T*-tests.

#### b) Self-emotion evaluation test

Using SAM, subjects were asked to rate valence, arousal, and dominance (V-A-D) on a scale of -4 to 4, with 0 as neutral: pleasure (+4) – displeasure (−4) for valence, excited (+4) – calm (−4) for arousal, and submissive (+4) – dominant (−4) for dominance according to Bradley and Lang [25] Subjects responded using a keyboard.

#### c) Statistical analysis with representative value of 16 stimuli

For the first analysis, we used the average score for sixteen stimuli of one category of expression as the representative score of each subject. We found large variance in individual subjects' scores for the 16 pictures within the same expression category. We therefore normalized the raw scores to z-scores. The raw-score (x) for each stimulus of every participant was normalized to the z-score (x′) as follows:
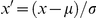
Here, μ indicates the mean, and σ is the standard deviation of the scores of all subjects (n = 122) for one stimulus regardless of genotype. We then calculated the mean (representative) z-score of each category of expression for individual subjects. Using this mean z-score, we performed three-way ANOVA (2 genotypes×3 emotions (V-A-D)×4 facial expressions) for the first statistical analysis, with two-way ANOVA for the post-hoc tests. Statistical analysis was performed using both raw data and the normalized data for most of the analysis unless stated.

#### d) Statistical tests with scores for individual stimuli in each expression category

The above statistical analysis was performed using averaged z-scores or averaged raw scores of 16 photographs of each expression category. We then compared the raw scores for each of the 16 stimuli of one category of facial expression between the 2 genotypes, by applying two-way ANOVA (2 genotypes×16 stimuli) in each combination of V-A-D×4 expressions (e.g. Anger/Dominance etc.) with consideration of the fact that the above averaged representative scores do not contain information about the level of emotional strength for each photograph.

#### e) Plot in 2-dimensional affective space

The mean valence and arousal rating of each genotype group were plotted in the two-dimensional affective space in order to compare them with the results of Bradley and Lang [26], and Lang and Davis [27]. In this plot, correlation coefficients were calculated in the Valence–Arousal space using the mean raw score for 16 stimuli for each genotype.

### Personality Tests

The Temperament and Character Inventory (TCI) [28] and Maudsley Personal Inventory (MPI) [29] were used for the personality tests.

MPI includes two categories; Extraversion score (E) and Neuroticism score (N). Lie score (L) was checked to verify consistency of their answers.

TCI includes seven categories; Novelty Seeking (NS), Harm Avoidance (HA), Reward Dependence (RD), Persistence (P), Self Directedness (SD), Cooperativeness (C), and Self Transcendence (ST). The genotype difference was tested by two-sampled T test for each category.

## Results

### Polymorphism in Human NPBWR1 Gene Affects Receptor Function

The human *NPBWR1* gene has a frequent single nucleotide polymorphism at nucleotide 404 (SNP rs33977775) in the coding region (404A>T). We genotyped 678 persons and found that 529 were 404AA (position 404 in both alleles of *NPBWR1* is A), 142 were 404AT (position 404 in one allele is replaced by T), and seven were 404TT (position 404 in both alleles of *NPBWR1* is replaced by T). Importantly, this polymorphism causes an amino acid substitution (Y135F) within the highly conserved DRY motif of G-protein-coupled receptors at the junction of the third transmembrane domain and second intracellular loop, which has been shown to be important for G-protein coupling. Mutations of residues within this motif usually abolish or severely impair receptor function [30], [31], suggesting the possibility that SNP rs33977775 affects human NPBWR1 function and behaviors that involve the amygdala.

**Figure 1:**
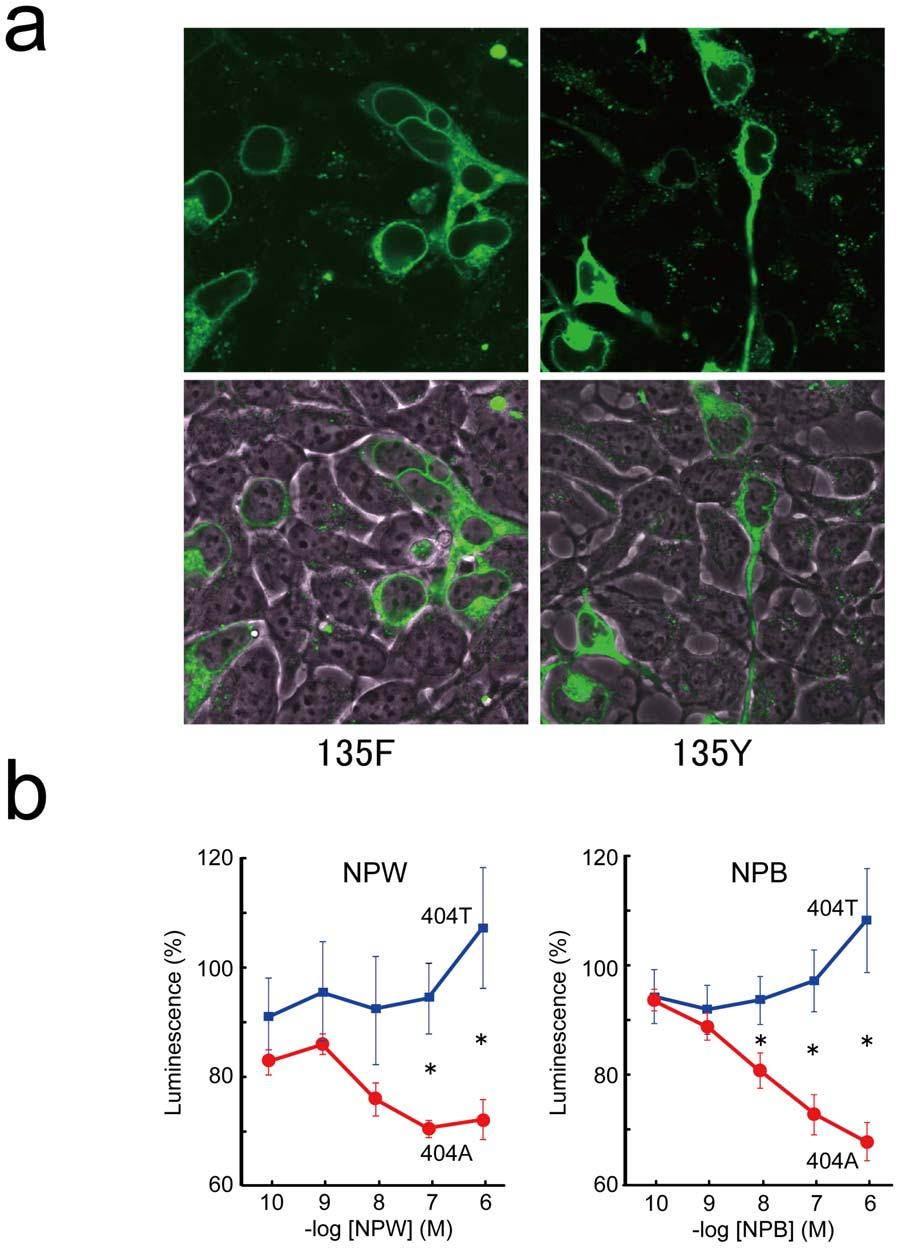
Effects of Polymorphism in Human NPBWR1 Gene. (A) Expression of NPBWR1::GFP (135F) (left) and NPBWR1::GFP (135Y) (right) in HEK293T cells. Receptor cDNAs were transfected and GFP expression was monitored by a laser confocal fluorescent microscope (FV1000, Olympus, Japan). Lower panels are high power views of rectangular regions in corresponding upper panels. (B) NPW- (left) or NPB- (right) dependent inhibition of forskoklin-induced accumulation of cellular cAMP was assessed in HEK293 cells transiently transfected with *NPBWR1 (404T)* (135F) (n = 6) or *NPBWR1 (404A)* (135Y) (n = 6) along with pGlosensor cAMP plasmid. cAMP levels were monitored by luciferase activity using Glo-sensor system (Promega). The effect seen in *NPBWR1 (404T)*-expressing cells was significantly weaker. Data are mean ± SD. *indicates *p*<0.05.

The level of expression and subcellular localization of each receptor were similar in the *NPBWR1* 404A and *NPBWR1* 404T constructs, judging from fluorescent intensities observed by laser confocal microscopy (Figure 1A). However, the amino acid change (Y135F) appreciably impaired the receptor function as assessed in transfected HEK293 cells (Figure 1B). We observed dose-dependent inhibition of cAMP accumulation by the addition of neuropeptide W or B in HEK293 cells expressing human *NPBWR1* with adenine in position 404 (*NPBWR1* 404A). However, this inhibitory effect on cAMP accumulation was significantly reduced in HEK293 cells expressing *NPBWR1* 404T both with NPW (−log 7M; *T_10_* = 2.53, *p* = 0.03, −log 6M; *T_10_* = 3.61, *p* = 0.005), and with NPB (−log 7M; *T_10_* = 2.90, *p* = 0.02, −log 6M; *T_10_* = 3.96, *p* = 0.005).

This observation, along with the aforementioned abnormality seen in *Npbwr1^−/−^* mice [21], suggested the possibility that the loss-of-function SNP (404AT) in *NPBWR1* might change NPBWR1 function in the amygdala and other regions, and thus affect human behavior. We therefore performed behavioral tests in subjects with these 2 genotypes.

### Behavioral Study

#### a) Genotypes

The details of the genetic profiles, sex, and age of the subjects are summarized in Table 1A. Genotyping revealed that the proportion of this Japanese sample with the 404AA genotype of the *NPBWR1* gene (position 404 in both alleles of *NPBWR1* is A) was 71.3%, the proportion with 404AT (position 404 in one allele is replaced by T) was 25.3%, and the proportion with 404TT (position 404 in both alleles of *NPBWR1* is replaced by T) was 3.4% (Table 1A). Because the total number of subjects with the 404TT genotype was small, this group was excluded from the statistical analysis. To exclude influences other than the SNP difference, we tested sex and age bias in each experiment. In the facial emotion identification and evaluation tests, 122 subjects participated, and no statistically significant difference was observed between the two genotype groups by sex (*χ^2^*_1_ = 0.15, *p* = 0.70) or age (*T_120_* = −0.80, *p* = 0.42). One hundred and fourteen subjects participated in the TCI personality test. No statistically significant difference was observed between the two genotype groups by sex (*χ^2^*_1_ = 0.10, *p* = 0.75) or age (*T_112_* = −1.14, *p* = 0.26). Additionally, 108 subjects participated in MPI. There was no statistically significant difference between the two groups by sex (*χ^2^*_1_ = 0.53, *p* = 0.47) or age (*T_106_* = 1.47, *p* = 0.15). These results indicate that the observed differences according to SNP types were not caused by sex or age bias.

**Table 1:** Genotype, age and sex of subjects.

A. all subjects								
NPBWR1	Total	(%)	Male	(%)	Female	(%)	Age y-old	
							Mean	SD
**404AA**	127	71.3	72	40.4	55	30.9	20.91	1.67
**404AT**	45	25.3	27	15.2	18	10.1	21.29	2.55
**404TT**	6	3.4	1	0.6	5	2.8	22.17	3.49
**Total**	178	-	100	56.2	78	43.8	21	2

B. subjects participated in viewing emotional faces				Age y-old	
NPBWR1	Total	Male	Female	Mean	SD
**404AA**	88	51	37	21.1	1.6
**404AT**	34	21	13	21.4	2.8
**Total**	122	72	50	21.2	2

C. subjects participated in TCI				Age y-old	
NPBWR1	Total	Male	Female	Mean	SD
**404AA**	82	46	36	21	1.5
**404AT**	32	19	13	21.5	2.9
**Total**	114	65	49	21.2	2

D. subjects participated in MPI				Age y-old	
NPBWR1	Total	Male	Female	Mean	SD
**404AA**	82	47	35	21.2	1.6
**404AT**	26	17	9	21.9	3.1
**Total**	108	64	44	21.4	2.1

Subjects with 404TT was not used for analysis due to their small number (n = 4). Neither Pearson's χ^2^ test for the sex distribution nor two-tailed *t*-test for the age distribution showed a significant difference (*p*>0.05).

We also compared the ratios of 404AT and 404AA according to the subjects' major fields; Humanities (404AA = 4, 404AT = 2), Social and International Studies (404AA = 7, 404AT = 3), Human Science (404AA = 5, 404AT = 2), Life and Environmental Sciences (404AA = 19, 404AT = 4), Science and Engineering (404AA = 20, 404AT = 7), Informatics (404AA = 5, 404AT = 4), Medical Sciences (Medicine, Medical Technology, Nursing) (404AA = 17, 404AT = 10), Physical Education (404AA = 8%, 404AT = 1%), and Art and Design (404AA = 3, 404AT = 1). We compared the ratios of 404AA and 404AT in each group and tested by χ^2^ test, and found no statistically significant difference in their ratios (χ^2^_8_ = 5.05, *p* = 0.75).

#### b) Error rates of facial emotion identification

No significant main effect on error rates for identifying facial expressions was observed among all categories between the two genotypes (*F*_3,360_ = 0.58, *p* = 0.45) or for the interaction (*F*_3,360_ = 1.21, *p* = 0.30), whereas a significant main effect was observed among the four expressions (*F*_1,120_ = 7.41, *p*<0.01) (Table 2A). Furthermore, error rates for mistaking one expression for another were not significantly different among any combination of two emotional expressions (Table 2B). Therefore, the difference in self-emotion evaluation described in the following sections was not due to failure to identify facial expressions.

**Table 2:** Genotype difference in error rates of facial expression identification.

A. Genotype difference in error rates				
Error Type	% of Errors: Mean (SD)		*T*(two tailed)	*p*
	404AA (n = 88)	404AT (n = 34)		
Anger	8.38 (12.78)	10.11 (9.36)	−0.72	0.47
Fear	12.07 (14.67)	7.90 (9.14)	1.54	0.13
Happy	5.75 (9.15)	4.78 (8.71)	0.53	0.59
Neutral	13.14 (14.28)	11.76 (12.94)	0.49	0.63

B. Genotype difference in error rates of mistaking one expression for another				
Error Type	% of Errors: Mean (SD)		*T*(two tailed)	*p*
	404AA (n = 88)	404AT (n = 34)		
Mistaking Anger for Fear	5.54 (9.82)	5.33 (7.24)	−0.13	0.90
for Happy	0.28 (1.31)	0.55 (1.80)	0.79	0.43
for Neutral	2.56 (6.04)	4.23 (4.80)	1.60	0.11
Mistaking Fear for Anger	6.89 (9.57)	5.33 (6.37)	−1.04	0.30
for Happy	0.28 (1.31)	0.18 (1.07)	−0.43	0.67
for Neutral	4.90 (9.59)	2.39 (5.76)	−1.77	0.08
Mistaking Happy for Anger	0.64 (3.00)	0.74 (2.56)	0.18	0.86
for Fear	0.07 (0.67)	0.37 (1.49)	1.12	0.27
for Neutral	5.04 (8.03)	3.68 (6.18)	−1.00	0.32
Mistaking Neutral for Anger	9.87 (12.01)	7.90 (9.27)	−0.96	0.34
for Fear	2.06 (4.42)	3.13 (9.14)	0.65	0.52
for Happy	1.21 (3.53)	0.74 (2.56)	−0.82	0.42

Although we calculated the error rates for each expression and for combinations of two expressions (e.g. mistaking fear for anger), no significant difference was observed between the two SNPs.

#### c) Self-emotion evaluation

We found a statistically significant interaction effect between genotypes and emotion factor (V-A-D) by three-way ANOVA (*F*_2,240_ = 4.06, *p* = 0.02) using normalized scores, whereas no significant main effect was observed for genotypes (*F*_1,120_ = 0.002, *p* = 0.96), V-A-D (*F*_2,240_ = 0.80, *p* = 0.45) or expressions (*F*_3,360_ = 0.21, *p* = 0.89) (Table S1). None of the other interactions, genotype×facial expression (*F*_3,360_ = 1.07, *p* = 0.36), V-A-D×facial expression (*F*_6,720_ = 0.13, *p* = 0.99), or genotype×V-A-D×facial expression (*F*_6,720_ = 0.65, *p* = 0.69), was significant.

**Figure 2:**
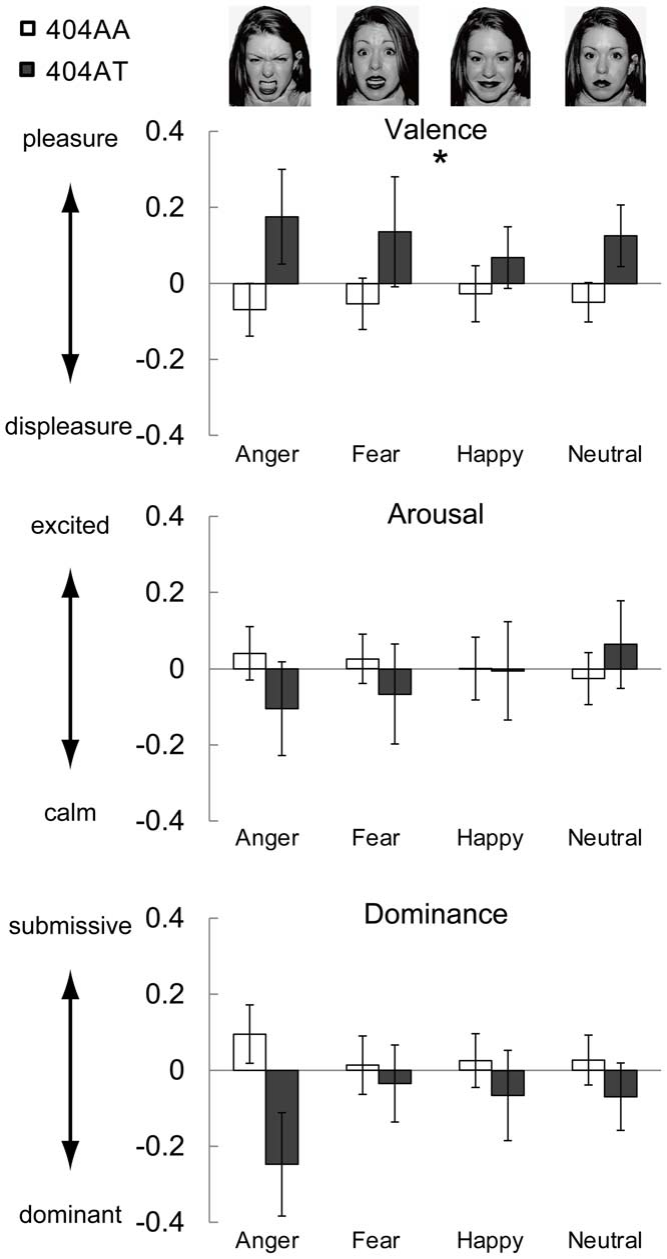
Self-emotional evaluation scores according to three factor theory. Normalized Z-scores of 2 groups are shown as mean + SE. White bar: 404A/A (n = 88), gray bar: 404A/T (n = 34). Score 0 is the mean of all subjects (n = 122). A statistically significant difference between 2 groups was observed in the main effect of valence scores (404AA>404AT). Statistical details are shown in Table S1.

As there was an interaction for genotype×V-A-D, we tested whether the normalized scores for valence, arousal, and dominance differed according to facial expression by two-way ANOVA. There was a statistically significant main effect of genotype on valence (*F*_1,120_ = 5.98, *p* = 0.02). No significant main effect of genotype on arousal (*F* = 0.19, *p* = 0.66) or dominance (*F* = 2.54, *p* = 0.11) was observed (Figure 2 and Table S1). In order to confirm the results using z-scores, we analyzed the data using raw scores. We observed similar results to those with normalized scores; a statistically significant interaction between genotype and V-A-D was observed by three-way ANOVA (*F*_2,240_ = 3.63, *p* = 0.03), and a significant main effect of genotype was observed only for valence by post-hoc two-way ANOVA (*F*_1,120_ = 5.66, *p* = 0.02) (Table S2). The results indicated that 404AT subjects tended to perceive any of the four expressions generally in more pleasant terms than did the 404AA subjects. In other words, 404AT subjects had more positive attitudes toward social interactions than did 404AA subjects.

**Figure 3:**
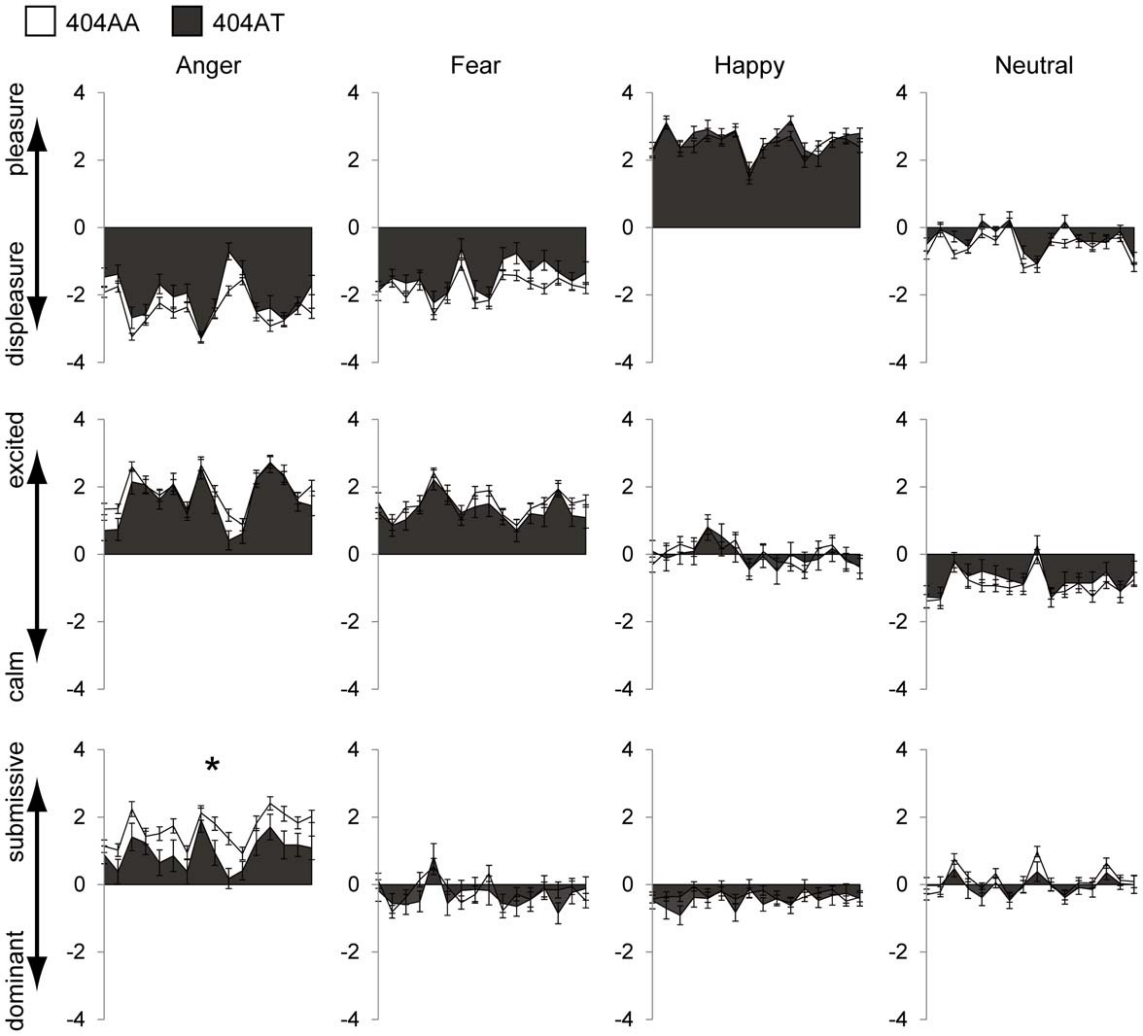
Self-emotional evaluation raw scores for 16 individual stimuli are plotted for each combination of V-A-D×Expression. Main effect was observed only in the domain of Dominant×Anger between 404AA (n = 88, white) and 404AT (n = 34, gray) (p = 0.03). Vertical axis shows mean + SE and horizontal axis shows 16 stimuli. * indicates *p*<0.05.

In addition to statistical analysis with representative values, analysis using raw scores for the 16 individual stimuli in each expression category showed a significant difference between the 2 genotypes. By two-way ANOVA (2 genotypes×16 stimuli) for each combination of [facial expression×V-A-D], a significant main effect of genotype was observed for angry expression with dominance rating (*F*_1,120_ = 5.11, *p* = 0.03). Thus, 404AT subjects showed less submissiveness to angry faces compared to 404AA subjects (mean ± SD; 404AA = 1.65±1.43, 404AT = 0.98±1.58) (Figure 3). No other combination of [facial expression and V-A-D] showed a significant main effect between the two genotypes, although we observed a marginally significant (*p*<0.1) difference in angry expression with valence rating (*F*_1,120_ = 3.45, *p* = 0.07) and in neutral expression with valence rating (*F*_1,120_ = 3.44, *p* = 0.07), which were consistent with the analysis with representative values. (As expected, a significant main effect of stimulus was observed for all combinations.) Interaction between genotype and stimulus was observed only for angry face with valence rating (*F*_15,1800_ = 2.42, *p*<0.01).

#### d) Correlation between Valence and Arousal

**Figure 4:**
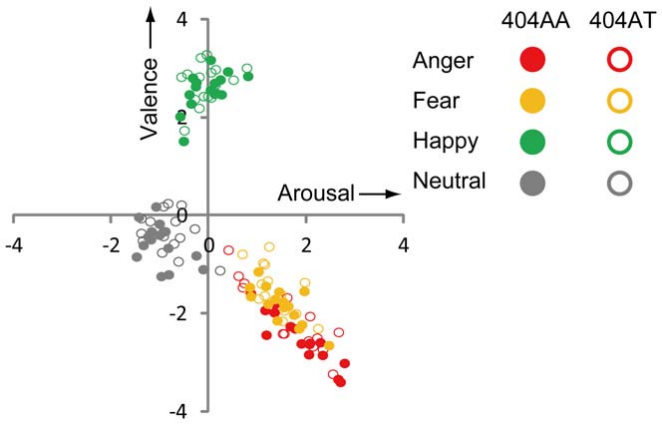
Plots in 2-dimensional affective space of valence-arousal. Each point indicates average raw scores of all 404AA subjects (n = 88, filled circles) or all 404AT subjects (n = 34, open circles) for each stimulus picture. The plot was made according to the methods of Bradley and Lang [26], and Lang and Davis [27].

In order to confirm that facial stimuli could be useful for the evaluation of emotion, we plotted the two-dimensional affective space between valence and arousal according to the methods by Bradley and Lang [22], [26], and Lang and Davis [27] using average raw scores for each category of facial expression. The distribution of scores in the valence-arousal space showed a similar distribution to that in previous studies [26], [27] (Figure 4). The unique feature of using facial expressions as stimuli is that each expression is distributed in a certain quadrant as a cluster. Clear correlation was observed for anger (*r* = −0.92, *p*<0.01 for 404AA and *r* = −0.89, *p*<0.01 for 404AT) and fear (*r* = −0.76, *p*<0.01 for 404AA and *r* = −0.61, *p* = 0.01 for 404AT) in the same quadrant, whereas for happy stimuli, there was a significant correlation only for 404AA (*r* = −0.59, *p* = 0.02) but not for 404AT (*r* = 0.34, *p* = 0.20). With neutral, no correlation was observed for 404AA (r = −4.41, *p* = 0.09) or for 404AT (r = −0.36, *p* = 0.17).

### Personality Tests

MPI showed no significant difference between the two genotypes in either E score (*T*_106_ = 0.33, *p* = 0.75) or N score (*T*_106_ = −0.26, *p* = 0.80) in 108 subjects (Table S3A). Therefore, NPBWR1 does not seem have an obvious effect on the personality factor of extraversion or introversion.

In TCI, among seven categories, only RD showed a significant difference between 404AA (14.98±3.69) and 404AT (13.34±4.01) (*T*_114_ = 2.07 *p* = 0.04) (Table S3B). RD is manifest as 3 subcategories; sentimentality (RD1), social attachment (RD3) and dependence on approval of others (RD4) [28]. When genotype difference was tested in the 3 subcategories separately, none of the subcategories showed a significant difference between the two genotypes.

## Discussion

The results of this study demonstrated that the genotype difference caused by an SNP of NPBWR1 changed the receptor function of this molecule at the cellular level, and, at the behavioral level, could influence personality difference in the context of social interaction. SAM was originally developed for measuring emotional responses that involve the autonomic nervous system. A stranger's face may not really be an emotionally engaging stimulus compared to other stronger stimuli in the sense that observers feel a strong emotional response themselves. Even under such limitation, in this experiment, SAM was useful for evaluating subtle emotional changes involved in social interaction. The results of correlation analysis between valence and arousal with a face stimulus, using the methods by Bradley and Lang [26] and Lang and Davis [27], showed that facial stimuli could be useful for evaluating emotional response in a social context.

NPBWR1 was shown to be distributed in the amygdala, hippocampus and substantia nigra in the human brain, based on measurement of mRNA level in the post-mortem brain [12]. A more detailed anatomical study in rodents showed that the distribution of *Npbwr1* mRNA was restricted to particular cell groups including the CeA and the bed nucleus of stria terminalis, hypothalamus, hippocampus, and VTA [11], [13]. These regions are known to be involved in emotional responses including control of the autonomic nervous system and endocrine system, and in various reward-related behaviors.

### Valence and the amygdala

The results of the self-emotion evaluation test showed a difference between the *NPBWR1* 404AA and 404AT genotype groups only in scores of the valence factor, with no effect on arousal and dominance. It is plausible that the lower scores for valence in individuals with the 404AT genotype may merely imply their difficulties in identifying facial expressions. However, as error rates for mistaking one expression for another were not significantly different between the 404AA and 404AT groups, the results indicate that the identification process itself was not influenced by the genotypes.

Whether valence is coded in amygdala activity has been debated. Some papers have suggested that the amygdala might not be sensitive to the valence of a stimulus, that is, how negative or positive it is, but might mediate emotional arousal associated with highly unpleasant and pleasant stimuli [32]–[43]. Visual stimuli used to test the relationship between amygdala activity and emotion have often been more negative or positive than pictures used as reference stimuli. Therefore, the tight connection between stimulus-associated valence and arousal makes it difficult to dissociate neural groups involved in the processing of valence and arousal. However, recent studies that disassociated valence and arousal (stimulus intensity) showed that the amygdala was sensitive to the valence of pictures that were equal in arousal value [44]. Wright et al. [45] also reported that the left, but not the right, amygdala exhibited sustained differential valence responses, with higher activation to fearful than to happy faces in a repeated-presentation paradigm. In a similar fashion, a meta-analysis of 105 fMRI studies using facial expressions as stimuli revealed that stimuli with a negative valence (fearful face) showed higher activity in the amygdala than did those with a positive valence (happy face) [46]. Additionally, Kim et al. [47] reported that in subjects who rated surprised faces with more negative valence, higher activation of the right amygdala was observed. These results all support the notion that the amygdala codes valence information.

Thus, the decreased function of NPBWR1 in CeA might contribute to valence-based evaluation of facial emotion. Also, our finding that the 404AT group tended to perceive all facial expressions in a more positive fashion than did 404AA subjects could be due to altered function of the CeA as a result of decreased function of Npbwr1.

### Valence and other brain regions

In addition to the involvement of the amygdala, other brain mechanisms possibly contribute to valence rating. Posner et al. [48] suggested that the valence dimension may be represented by the dopamine system. A large body of evidence has established the role of the midbrain dopamine system, especially the VTA and substantia nigra compacta, which projects to several limbic and cortical regions including the nucleus accumbens, amygdala, hippocampus, and orbitofrontal cortex [49]–[51]. It is generally accepted that the activity of dopamine neurons encodes information about reward, reward prediction, and reward prediction errors (see [20] for review). The involvement of the reward system in pleasure-seeking has been shown by self-stimulation tasks involving the orbitofrontal cortex and ventral striatum in animal studies [52], [53]. Whether pleasure-seeking and valence represent the same human emotional dimension may be debatable; however, functional imaging studies have also indicated that information about emotional valence is represented in the reward system. Using odor stimuli that induce positive or negative feelings, Anderson et al. [54] demonstrated that the activity of the right medial orbitofrontal cortex, which has abundant dopamine projections, was higher for positive odors than for negative odors regardless of intensity. Salimpoor et al. [55] measured dopamine release when subjects felt pleasure listening to music using positron emission tomography with [^11^C] raclopride, which binds to dopamine D2 receptors. Their results showed that listening to pleasurable music compared to neutral music increased dopamine release in the caudate, putamen, and nucleus accumbens. Sharot et al. [56] also demonstrated that, in humans, administration of a precursor of dopamine (L-DOPA) enhanced expectations of future pleasure compared with a placebo (vitamin C). Thus, the emotional valence (pleasure-sensing) system and dopamine reward system seem to overlap, at least partially, and the effect of NPBWR1-SNP might modify the function of this system resulting in the difference in valence evaluation.

In the brains of mice, Tanaka et al. [13] detected NPBWR1 mRNA expression in VTA by in situ hybridization. They also showed that the mRNA of NPW, which is one of the ligands of NPBWR1, was expressed in VTA as well. Kitamura et al. [57] reported that for rat brains as well, NPW-immunoreactive cells were detected at VTA. In the postmortem human brain, Brezillon et al. [12] showed by RT-PCR that NPBWR1 mRNA was expressed in the substantia nigra. The expression of both the ligand and receptor in the same region could indicate the existence of an autoreceptor for NPW. However, as it has not been identified whether NPW and NPBWR1 are expressed in the same neurons; further research is needed to determine the role of this peptide in this region.

*Npbwr1* ^−/−^ mice showed impulsive contact with intruder mice, produced more intense approaches toward them, and had longer contact and chasing times [21]. It is possible that these abnormal behaviors were caused by inappropriate estimation of negative social values (i.e., intruder mice). Although we still have no direct evidence that NPBWR1 modulates the dopamine-related reward system, based on the tissue distribution and behavior of *Npbwr1* ^−/−^ mice, NPBWR1 might play a role in the reward system.

### Dominance to angry faces and the amygdala

Additional analysis using individual scores for the 16 stimuli in one category of expression revealed that 404AT subjects seemed to feel less submissive to angry faces compared to 404AA subjects. Generally, seeing angry faces may induce fear-related reactions in an observer, such as decreased ability to control that person (feeling that the other person dominates the subject), and lead to a state of high arousal and strong displeasure.

Recent studies suggest that amygdala activity is important for appropriate social interactions [58]. In monkeys with selective lesions of the bilateral central nucleus of amygdala, freezing behavior decreased when confronted by a human intruder [59]. In addition, a patient with bilateral amygdala lesion (Urbach-Wiethe disease) showed inappropriate social behavior and failed to maintain employment or marital relations [15]. Recently Kennedy et al. reported that healthy individuals showed amygdala activation upon close personal proximity, but patient S.M. lacked any sense of personal space [60]. In humans, taking a distance from others may derive from social fear as well as from consideration not to disturb others. In *Npbwr1* ^−/−^ mice, the shorter latency and persistent chasing of the intruder may relate to lack of fear of intruders. That 404AT subjects felt less domination than did 404AA subjects by an angry expression may derive from a decreased sense of fear, which is related to the amygdala.

### Personality difference depending on NPBWR1 genotypes

While we did not find any difference in the MPI test, in the TCI personality test, we observed a statistically significant difference in RD between the two NPBWR1 genotypes. RD is a heritable tendency believed to involve an intense response to social reward signals [3]. Individuals with higher RD are clinically characterized as eager to help and please others, warmly sympathetic, sentimental, and sensitive to social cues (sociable), whereas individuals with lower RD tend to respond to practical rewards such as money, but are insensitive and aloof to verbal social reinforcement signals. This result seems consistent with the results of facial stimuli. As 404AT subjects seem to be less sensitive to emotions of other people, even though they see angry people, they may care less (unconsciously) about the outcome of their behavior.

Our findings provide evidence that human genetic differences in NPBWR1 modulate emotional responses to facial expressions. Specific difference in response between 2 genotypes was found in valence evaluation, and in dominance rating in seeing angry faces. In our daily life, people show various emotional reactions to others depending on their personality, even in the same situation. NPBWR1 may provide part of the cause for such differences in reactions in social interactions.

## Supporting Information

Table S1 **Genotype difference in self-emotion evaluation by normalized score.** (**A**) By three-way ANOVA (Genotype×V-A-D×Expression), significant interaction was observed in Genotype×V-A-D (*p* = 0.02). (**B**) By two-way ANOVA (Genotype×Expression) for each V-A-D scale, main effect of genotype was observed only for Valence. SS: sum of squares, df: degrees of freedom, MS: mean square, *F*: *F* value.
(DOC)

Table S2 **Genotype effect on self-emotion evaluation by raw scores.** (**A**) Average scores of self-emotion evaluation. (**B**) By three-way ANOVA (Genotype×V-A-D×Expression), significant interaction was observed in Genotype×V-A-D. (*p* = 0.03). (**C**) By two-way ANOVA (Genotype×Expression) of each V-A-D scale, main effect of genotype was observed only for Valence. These results are consistent with analysis with normalized scores.
(DOC)

Table S3 **Genotype effect on personality tests.** (**A**) There was no statistically significant difference in MPI scores. (**B**) In TCI, only RD (reward dependence) score showed a significant difference between the two genotypes. However, none of the sub-categories showed a significant difference in RD (**C**).
(DOC)
