# Supplementary material for: A Single Nucleotide Polymorphism of the Neuropeptide B/W Receptor-1 Gene Influences the Evaluation of Facial Expressions
Source: PLoS One. 2012 Apr 24;7(4):e35390. doi: 10.1371/journal.pone.0035390 (PMC3335863; doi:10.1371/journal.pone.0035390)
Supplement: Table S1 — Genotype difference in self-emotion evaluation by normalized score. (A) By three-way ANOVA (Genotype×V-A-D×Expression), significant interaction was observed in Genotype×V-A-D (p = 0.02). (B) By two-way ANOVA (Genotype×Expression) for each V-A-D scale, main effect of genotype was observed only for Valence. SS: sum of squares, df: degrees of freedom, MS: mean square, F: F value. (DOC) [file pone.0035390.s001.doc]

**Table S1** Genotype difference in self-emotion evaluation by normalized score. (**A**) By three-way ANOVA (Genotype x V-A-D x Expression), significant interaction was observed in Genotype x V-A-D (*p* = 0.02). (**B**) By two-way ANOVA (Genotype x Expression) for each V-A-D scale, main effect of genotype was observed only for Valence. SS: sum of squares, df: degrees of freedom, MS: mean square, *F*: *F* value.

**A. three-way ANOVA of self-emotion evaluation**

| **Genotype(2) x V-A-D(3) x Expression(4)** | | | | | |
| --- | --- | --- | --- | --- | --- |
| **Source** | **SS** | **df** | **MS** | ***F*** | ***p*** |
| **Genotype** | 0.002 | 1 | 0.002 | 0.002 | 0.96 |
| **Error [Genotype]** | 95.817 | 120 | 0.798 |  |  |
| **V-A-D** | 1.025 | 2 | 0.513 | 0.8 | 0.45 |
| **Genotype × V-A-D** | 5.234 | 2 | 2.617 | 4.06 | **0.02*** |
| **Error [Genotype × V-A-D]** | 154.814 | 240 | 0.645 |  |  |
| **Expression** | 0.142 | 3 | 0.047 | 0.21 | 0.89 |
| **Genotype × Expression** | 0.727 | 3 | 0.242 | 1.07 | 0.36 |
| **Error [Genotype × Expression]** | 81.515 | 360 | 0.226 |  |  |
| **V-A-D × Expression** | 0.322 | 6 | 0.054 | 0.13 | 0.99 |
| **Genotype × V-A-D × Expression** | 1.642 | 6 | 0.274 | 0.65 | 0.69 |
| **Error [V-A-D × Expression]** | 301.483 | 720 | 0.419 |  |  |

**B. two-way ANOVA of self-emotion evaluation in each V-A-D scale.**

| **Valence** | | | | | |
| --- | --- | --- | --- | --- | --- |
| **Source** | **SS** | **df** | **MS** | ***F*** | ***p*** |
| **Genotype** | 3.035 | 1 | 3.035 | 5.98 | **0.02*** |
| **Error [Genotype]** | 60.946 | 120 | 0.508 |  |  |
| **Expression** | 0.055 | 3 | 0.018 | 0.05 | 0.99 |
| **Genotype x Expression** | 0.282 | 3 | 0.094 | 0.26 | 0.85 |
| **Error [Genotype × Expression]** | 128.95 | 360 | 0.358 |  |  |

| **Arousal** | | | | | |
| --- | --- | --- | --- | --- | --- |
| **Source** | **SS** | **df** | **MS** | ***F*** | ***p*** |
| **Genotype** | 0.15 | 1 | 0.15 | 0.19 | 0.66 |
| **Error [Genotype]** | 92.988 | 120 | 0.775 |  |  |
| **Expression** | 0.151 | 3 | 0.05 | 0.14 | 0.94 |
| **Genotype x Expression** | 0.772 | 3 | 0.257 | 0.7 | 0.55 |
| **Error [Genotype × Expression]** | 131.91 | 360 | 0.366 |  |  |

| **Dominance** | | | | | |
| --- | --- | --- | --- | --- | --- |
| **Source** | **SS** | **df** | **MS** | ***F*** | ***p*** |
| **Genotype** | 2.05 | 1 | 2.05 | 2.54 | 0.11 |
| **Error [Genotype]** | 96.697 | 120 | 0.806 |  |  |
| **Expression** | 0.257 | 3 | 0.086 | 0.25 | 0.86 |
| **Genotype x Expression** | 1.314 | 3 | 0.438 | 1.29 | 0.28 |
| **Error [Genotype × Expression]** | 122.14 | 360 | 0.339 |  |  |
