# Supplementary material for: A Single Nucleotide Polymorphism of the Neuropeptide B/W Receptor-1 Gene Influences the Evaluation of Facial Expressions
Source: PLoS One. 2012 Apr 24;7(4):e35390. doi: 10.1371/journal.pone.0035390 (PMC3335863; doi:10.1371/journal.pone.0035390)
Supplement: Table S2 — Genotype effect on self-emotion evaluation by raw scores. (A) Average scores of self-emotion evaluation. (B) By three-way ANOVA (Genotype×V-A-D×Expression), significant interaction was observed in Genotype×V-A-D. (p = 0.03). (C) By two-way ANOVA (Genotype×Expression) of each V-A-D scale, main effect of genotype was observed only for Valence. These results are consistent with analysis with normalized scores. (DOC) [file pone.0035390.s002.doc]

**Table S2** Genotype effect on self-emotion evaluation by raw scores. (**A**) Average scores of self-emotion evaluation. (**B**) By three-way ANOVA (Genotype x V-A-D x Expression), significant interaction was observed in Genotype x V-A-D. (*p* = 0.03). (**C**) By two-way ANOVA (Genotype x Expression) of each V-A-D scale, main effect of genotype was observed only for Valence. These results are consistent with analysis with normalized scores.

**A. Average scores of self-emotion evaluation**

| **Valence** | | |
| --- | --- | --- |
|  | **Mean (SD)** | |
|  | **404AA (n=88)** | **404AT (n=34)** |
| Anger | -2.44 (0.94) | -2.07 (1.07) |
| Fear | -1.77 (1.02) | -1.47 (1.35) |
| Happy | 2.47 (0.97) | 2.60 (0.66) |
| Neutral | -0.54 (0.54) | -0.33 (0.52) |

| **Arousal** | | |
| --- | --- | --- |
|  | **Mean (SD)** | |
|  | **404AA (n=88)** | **404AT (n=34)** |
| Anger | 1.86 (1.05) | 1.63 (1.16) |
| Fear | 1.49 (0.94) | 1.33 (1.19) |
| Happy | 0.01 (1.58) | -0.01 (1.54) |
| Neutral | -0.91 (1.14) | -0.75 (1.19) |

| **Dominance** | | |
| --- | --- | --- |
|  | **Mean (SD)** | |
|  | **404AA (n=88)** | **404AT (n=34)** |
| Anger | 1.65 (1.43) | 0.98 (1.58) |
| Fear | -0.21 (1.47) | -0.31 (1.20) |
| Happy | -0.33 (0.90) | -0.45 (0.95) |
| Neutral | 0.11 (0.86) | -0.02 (0.73) |

**B. three-way ANOVA of self-emotion evaluation**

| **Genotype(2) x V-A-D(3) x Expression(4)** | | | | | |
| --- | --- | --- | --- | --- | --- |
| **Source** | **SS** | **df** | **MS** | ***F*** | ***p*** |
| **Genotype** | 0.177 | 1 | 0.177 | 0.081 | 0.78 |
| **Error [Genotype]** | 263.409 | 120 | 2.195 |  |  |
| **V-A-D** | 209.178 | 2 | 104.589 | 59.139 | < 0.01* |
| **Genotype × V-A-D** | 12.825 | 2 | 6.413 | 3.626 | **0.03*** |
| **Error [Genotype × V-A-D]** | 424.447 | 240 | 1.769 |  |  |
| **Expression** | 214.598 | 3 | 71.533 | 98.695 | < 0.01* |
| **Genotype × Expression** | 2.64 | 3 | 0.88 | 1.214 | 0.30 |
| **Error [Genotype × Expression]** | 260.923 | 360 | 0.725 |  |  |
| **V-A-D × Expression** | 1719.334 | 6 | 286.556 | 243.248 | < 0.01* |
| **Genotype × V-A-D × Expression** | 5.957 | 6 | 0.993 | 0.843 | 0.54 |
| **Error [V-A-D × Expression]** | 848.19 | 720 | 1.178 |  |  |

**C. two-way ANOVA of self-emotion evaluation** in each V-A-D scale.

| **Valence** | | | | | |
| --- | --- | --- | --- | --- | --- |
| **Source** | **SS** | **df** | **MS** | ***F*** | ***p*** |
| **Genotype** | 6.011 | 1 | 6.011 | 5.66 | **0.02*** |
| **Error [Genotype]** | 127.459 | 120 | 1.062 |  |  |
| **Expression** | 1326.331 | 3 | 442.11 | 592.894 | < 0.01* |
| **Genotype x Expression** | 0.82 | 3 | 0.273 | 0.367 | 0.78 |
| **Error [Genotype × Expression]** | 268.445 | 360 | 0.746 |  |  |

| **Arousal** | | | | | |
| --- | --- | --- | --- | --- | --- |
| **Source** | **SS** | **df** | **MS** | ***F*** | ***P*** |
| **Genotype** | 0.37 | 1 | 0.37 | 0.159 | 0.69 |
| **Error [Genotype]** | 279.198 | 120 | 2.327 |  |  |
| **Expression** | 428.915 | 3 | 142.972 | 116.531 | < 0.01* |
| **Genotype x Expression** | 2.174 | 3 | 0.725 | 0.591 | 0.62 |
| **Error [Genotype × Expression]** | 441.683 | 360 | 1.227 |  |  |

| **Dominance** | | | | | |
| --- | --- | --- | --- | --- | --- |
| **Source** | **SS** | **df** | **MS** | ***F*** | ***P*** |
| **Genotype** | 6.622 | 1 | 6.622 | 2.826 | 0.1 |
| **Error [Genotype]** | 281.2 | 120 | 2.343 |  |  |
| **Expression** | 178.686 | 3 | 59.562 | 53.742 | < 0.01* |
| **Genotype x Expression** | 5.602 | 3 | 1.867 | 1.685 | 0.17 |
| **Error [Genotype × Expression]** | 398.985 | 360 | 1.108 |  |  |
