# Supplementary material for: A Single Nucleotide Polymorphism of the Neuropeptide B/W Receptor-1 Gene Influences the Evaluation of Facial Expressions
Source: PLoS One. 2012 Apr 24;7(4):e35390. doi: 10.1371/journal.pone.0035390 (PMC3335863; doi:10.1371/journal.pone.0035390)
Supplement: Table S3 — Genotype effect on personality tests. (A) There was no statistically significant difference in MPI scores. (B) In TCI, only RD (reward dependence) score showed a significant difference between the two genotypes. However, none of the sub-categories showed a significant difference in RD (C). (DOC) [file pone.0035390.s003.doc]

**Table S3** Genotype effect on personality tests. (**A**) There was no statistically significant difference in MPI scores. (**B**) In TCI, only RD (reward dependence) score showed a significant difference between the two genotypes. However, none of the sub-categories showed a significant difference in RD (**C**).

**A. Genotype difference in MPI**

|  | **Mean (SD)** | |  |  |
| --- | --- | --- | --- | --- |
| **Score** | **404AA (n=82)** | **404AT (n=26)** | ***T*(two tailed)** | ***p*** |
| Extraversion score | 28.3 (-12.9) | 27.54 (-9.51) | 0.33 | 0.75 |
| Neuroticism score | 24.16(-12.61) | 24.73 (-8.87) | -0.26 | 0.80 |

**B. Genotype difference in TCI**

|  | **Mean (SD)** | |  |  |
| --- | --- | --- | --- | --- |
| **Score** | **404AA (n=82)** | **404AT (n=32)** | ***T*(two tailed)** | ***p*** |
| NS total | 21.95 (-4.8) | 22.91 (-6.33) | -0.87 | 0.39 |
| HA total | 18.45 (-6.93) | 16.44 (-6.94) | 1.39 | 0.17 |
| RD total | 14.98 (-3.69) | 13.34 (-4.01) | 2.07 | **0.04*** |
| P total | 5.00 (-2.02) | 4.91 (-2.16) | 0.22 | 0.83 |
| SD total | 24.80 (-6.81) | 26.13 (-6.36) | -0.95 | 0.35 |
| C total | 27.30 (-6.04) | 27.47 (-6.35) | -0.13 | 0.90 |
| ST total | 11.85 (-4.75) | 12.69 (-6.3) | -0.77 | 0.45 |

**C. RD sub-scores in TCI**

|  | **Mean (SD)** | |  |  |
| --- | --- | --- | --- | --- |
| **Score** | **404AA (n=82)** | **404AT (n=32)** | ***T*(two tailed)** | ***p*** |
| RD1 | 5.71 (-1.92) | 5.31 (-1.91) | 0.99 | 0.33 |
| RD3 | 4.95 (-2.14) | 4.13 (-1.93) | 1.90 | 0.06 |
| RD4 | 4.32 (-1.3) | 3.91 (-1.51) | 1.45 | 0.15 |
